# Morphometric Predictors of Penile Length Increase After Division of its Suspensory Ligament

**DOI:** 10.1007/s00266-023-03837-7

**Published:** 2024-01-29

**Authors:** Márcio Ramos, Alice Varanda Pereira, Leonardo Silva, Ana Rita Inácio, Ivo Álvares Furtado

**Affiliations:** 1 Lisbon Faculty of Medicine, Lisbon, Portugal; 2 Local Health Unit of Santa Maria, Lisbon, Portugal; 3 LMR Plastic Surgery, Lisbon, Portugal; 4 National Institute of Legal Medicine and Forensic Sciences, Lisbon, Portugal; 5 Department of Medicine (Anatomy), Faculty of Life Sciences, University of Madeira, Funchal, Portugal

**Keywords:** Anatomy, Penis, Dissection, Elective surgical procedures, Esthetics, Sexuality

## Abstract

**Introduction:**

Division of the suspensory ligament of the penis has emerged as a popular surgical approach for penile lengthening, but accurate preoperative predictions of lengthening outcomes remain elusive. This study aimed to identify readily measurable anatomical parameters associated with post-ligamentolysis penile length gain, facilitating more reliable preoperative estimations.

**Methods:**

An experimental cross-sectional study was performed on 16 adult cadavers. Data collected before dissection included: age at death, ethnicity, height, length of the penis before dissection and width of the suspensory ligament of penis. Following the complete dissection of the suspensory ligament of penis, the depth of the pubic symphysis and the penile length after the procedure were measured. The absolute and relative length differences pre- and post-ligamentolysis were calculated. Correlation coefficients were used to study relations between these variables.

**Results:**

Penile length increased uniformly after complete division of the suspensory ligament (average gain: 26.38 mm, SD = 14.83 mm; range 4–60 mm). Pearson correlation revealed a significant negative correlation between pre-ligamentolysis penile length and post-ligamentolysis increase (*r* = − 0.601; *p* = 0.014), suggesting greater gains in individuals with shorter pre-ligamentolysis lengths. Age, ligament width, and pubic arch depth showed no significant correlations. Ethnicity did not impact post-ligamentolysis length increase (*t* = − 0.135; *p* = 0.894).

**Conclusions:**

This study highlights the potential to predict penile length gain post-ligamentolysis through measurable anatomical parameters. The ability to anticipate the outcome of this procedure could empower surgeons to provide informed counseling, potentially elevating patient satisfaction.

- An experimental cross-sectional study was performed to investigate the outcomes of penile lengthening surgery
- Penile lengthening was achieved in all subjects via complete dissection of the suspensory ligament of the penis
- Penile length increase may be predicted preoperatively using easily measurable anatomical parameters

**No Level Assigned:**

This journal requires that authors assign a level of evidence to each submission to which Evidence-Based Medicine rankings are applicable. This excludes Review Articles, Book Reviews, and manuscripts that concern Basic Science, Animal Studies, Cadaver Studies, and Experimental Studies. For a full description of these Evidence-Based Medicine ratings, please refer to the Table of Contents or the online Instructions to Authors www.springer.com/00266

## Introduction

Concerns about penis size have been a persistent feature of male psychology throughout history [1–8]. In recent times, penile enlargement surgeries have gained considerable popularity [1, 9–12]. Notably, individuals seeking these procedures typically possess average-sized penises and are motivated exclusively by aesthetic considerations, devoid of underlying psychopathology [5, 7, 11, 13–17].

One of the earliest and most extensively investigated surgical techniques for penile lengthening emerged in 1971, when Kelley and Eraklis developed a method to elongate the phallus of infants with bladder exstrophy [18]. The cornerstone of this technique involved the release of the suspensory ligaments of the penis [18]. This approach was subsequently refined and adapted for aesthetic and reconstructive purposes in adults, often combined with ancillary procedures such as the V-Y skin advancement flap [3, 5, 8, 13, 19–23].

The release of the penile suspensory ligament in conjunction with a V-Y skin advancement flap has become the most frequently performed penile lengthening procedure. However, the outcomes of this procedure exhibit substantial variability and remain challenging to predict. Currently, there exist no validated methods to anticipate the surgical results, leaving patients in a state of uncertainty regarding the procedure's outcome—a summary of surgical outcomes from independent reports is presented in Table 1.

**Table 1:** Summary of the reports of penile length increase, in millimeters, across studies performed by different authors. Hyphens indicate data that were not disclosed in the articles

	Range (Max.–Min.)	Mean (Std. Deviation)
[25]	–	35 (13)
[19]	–	13 (9)
[20]	33 (15–48)	20.42 (–)
[29]	30 (35–65)	–
[28]	65 (26–91)	–

Moreover, as a surgical intervention, this procedure entails inherent risks for patients, as outlined in Table 2. Understanding individual predicted outcomes is paramount for enabling patients to make informed decisions and for surgeons to guide patient care and decision-making.

**Table 2:** Summary of the possible complications of the surgical dissection of the suspensory ligament of penis [3–5, 15, 20, 22, 23, 30–32]

Possible complications of the surgical dissection of the suspensory ligament of penis
Lesions of the neurovascular bundles of the penis
Bulging of the penoscrotal transition
Opening of the penopubic angle, with horizontalization of the erect penis
Penile instability
Paradoxical penile shortening due to reattachment of the corpora in a more posterior position in the pubic symphysis
Hypertrophic wound scarring
Deep and superficial infections
Disfiguring advancement of suprapubic hairy skin
Wound dehiscence
Non resolving hematoma
Temporary erectile dysfunction

In this study, we aim to identify correlations between readily assessable morphometric parameters, including height, initial penile length, length of the suspensory ligament (measured by the depth of the pubic symphysis), width of the suspensory ligament proper, and the increase in length of the mobile flaccid penis. These correlations could serve as a decision-making tool for both surgeons and patients when considering this surgical procedure.

**Fig. 1:**
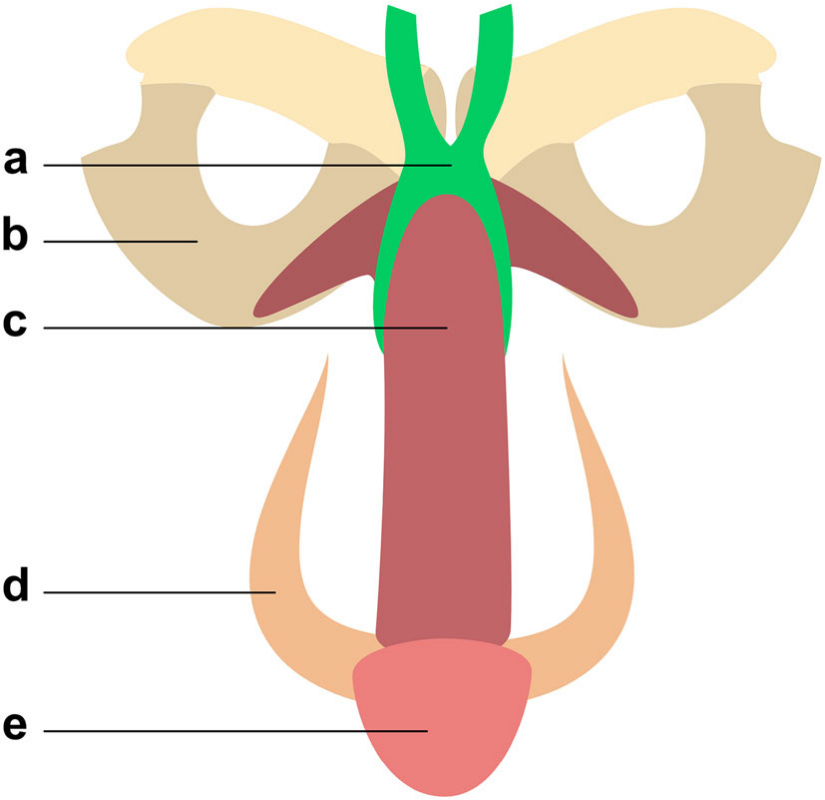
Coronal view of the fundiform ligament. Note that it embraces the circumference of the penis. Legend: **a** fundiform ligament; **b** hip bone; **c** corpora cavernosa; **d** scrotum; **e** glans penis

**Fig. 2:**
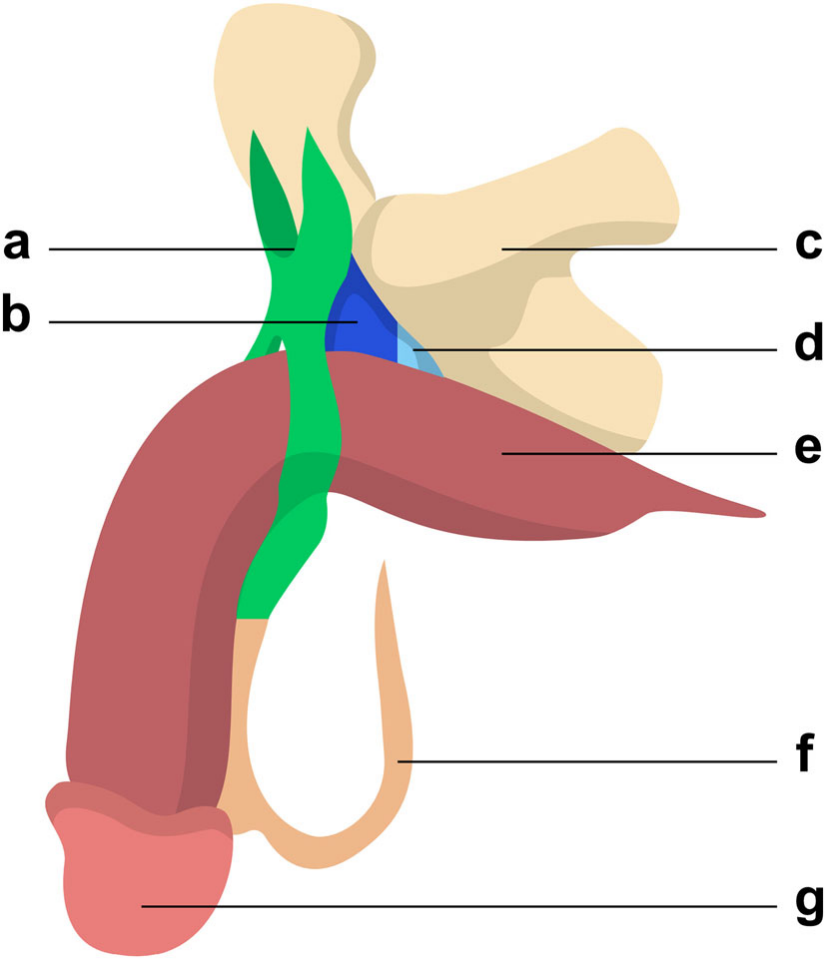
Anterolateral view of the suspensory ligamentous system of the penis. Note that, after encompassing the perimeter of the penis, the fundiform ligaments’ two bundles rejoin in the ventral aspect of the penis and constitute the superior part of the scrotal septum. Legend: **a** fundiform ligament; **b** suspensory ligament of penis; **c** hip bone; **d** inferior pubic ligament; **e** corpora cavernosa; **f** scrotum; **g** glans penis

**Fig. 3:**
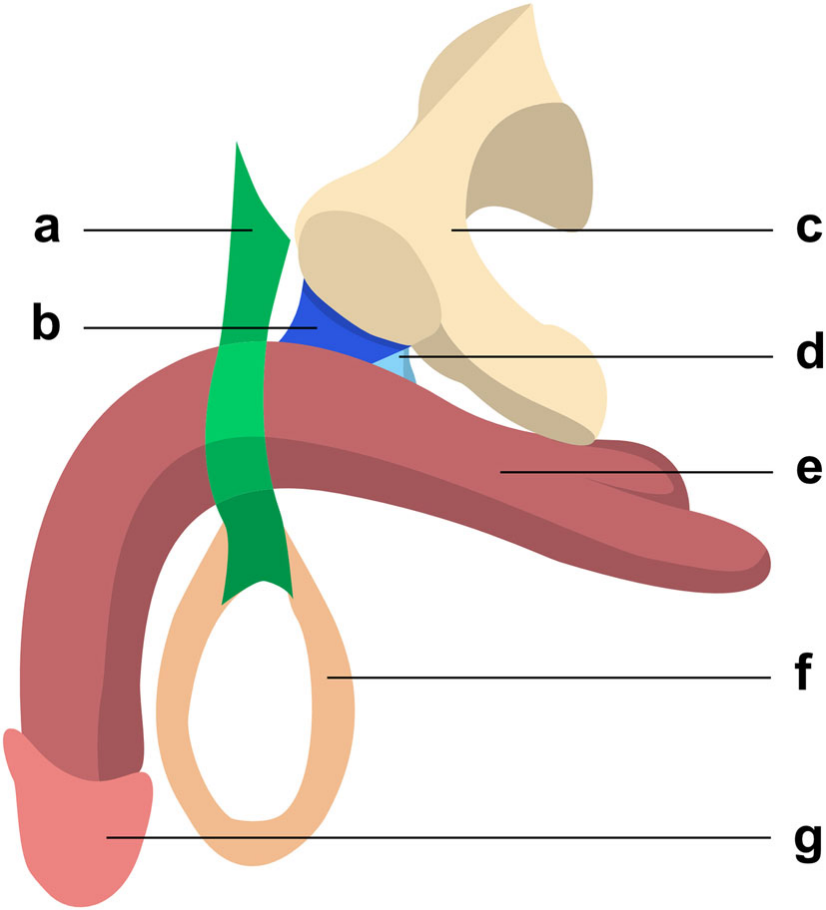
Sagittal view of the suspensory ligamentous system of the penis, offering another perspective on the relations between the different structures. Legend: **a** fundiform ligament; **b** suspensory ligament of penis; **c** hip bone; **d** inferior pubic ligament; **e** corpora cavernosa; **f** scrotum; **g** glans penis

**Fig. 4:**
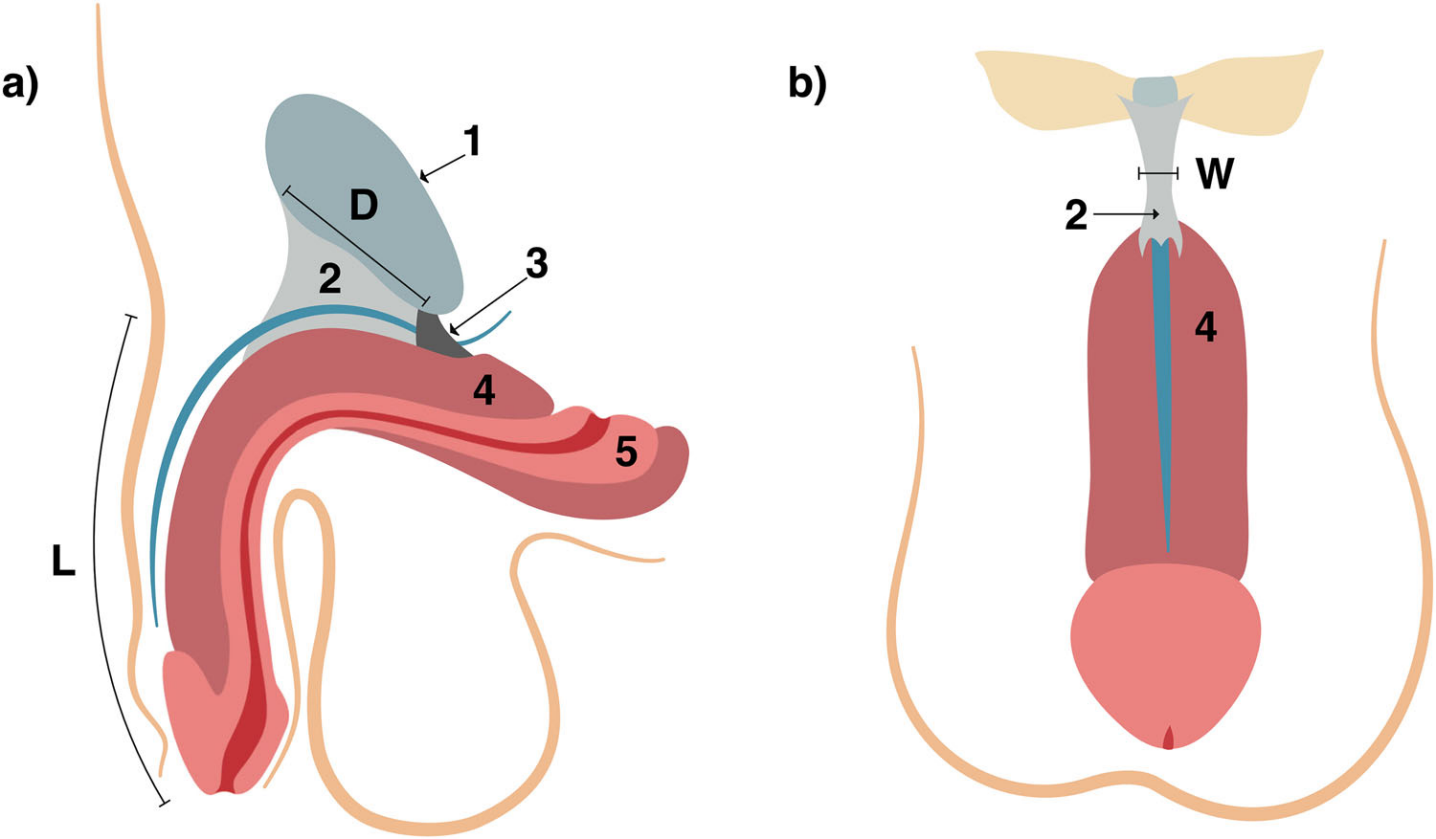
Sagittal (**a**) and coronal (**b**) views of the relevant structures and measurements: (1) pubic symphysis; (2) suspensory ligament the penis; (3) inferior pubic ligament; (4) corpora cavernosa; (5) corpus spongiosum. *D* represents the anteroposterior dimension (depth) of the pubic symphysis. *L* represents the length of the penis, measured from the pubo-penile junction to the tip of the glans. W represents the width of the suspensory ligament of penis

These parameters should also be readily measurable preoperatively, either through physical examination or non-invasive imaging techniques. The most relevant anatomical structures and corresponding measurements are highlighted in Figs. 1, 2, 3 and 4.

## Materials and Methods

An anatomical dissection study inspired by Littara's [12] surgical protocol for penile lengthening through division of the suspensory ligament and V-Y skin advancement flaps was conducted. This experimental cross-sectional study involved 16 penises from fresh adult cadavers. The dissections were performed by a senior plastic surgeon and anatomist, co-author of this study, with over a decade of experience in executing this procedure. The study started in October 2020, and was concluded in March 2021, at the National Institute of Legal Medicine and Forensic Sciences (INMLCF), Lisbon, Portugal. The study was authorized by the INMLCF's Department of Research, Training, and Documentation (DIFD) and adhered to the Declaration of Helsinki's guidelines.

### Data Collection

**Fig. 5:**
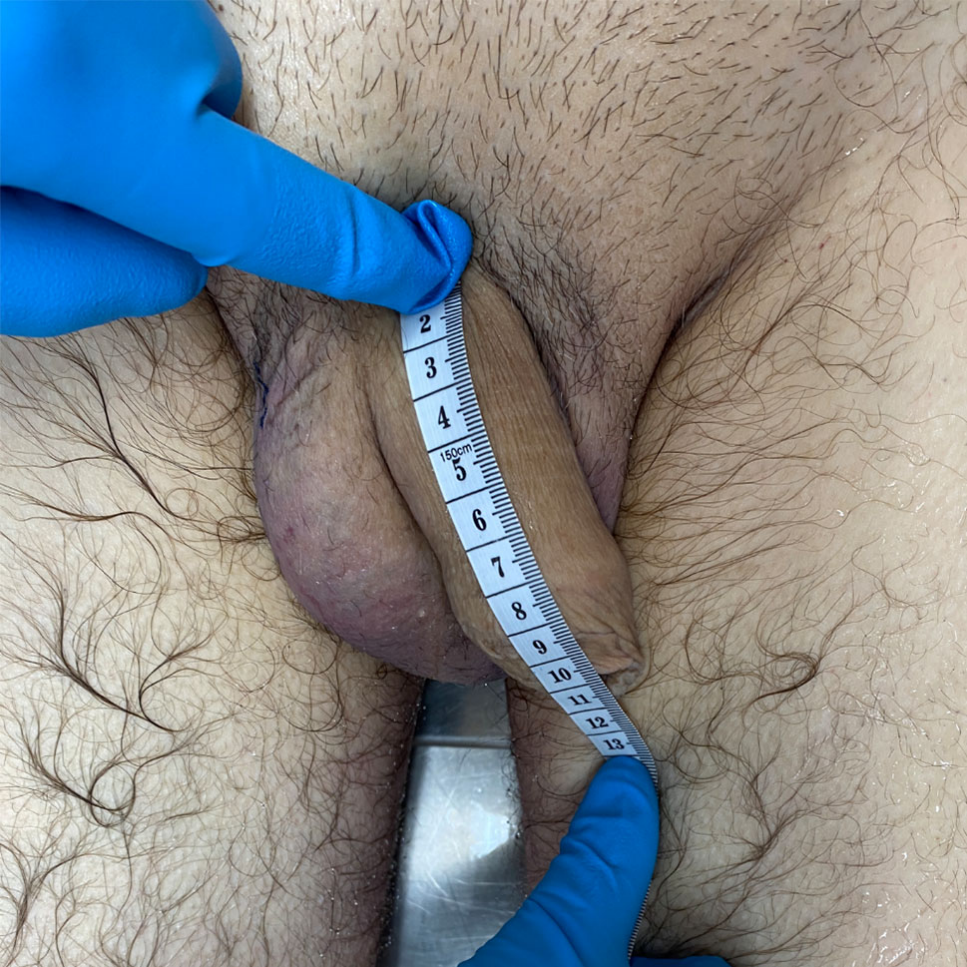
Measuring the initial penile length, using a disposable measuring tape

Essential information about each subject, including age at death and ethnicity (Asian, Black, Latino, or White), was meticulously recorded. Each subject's height was measured and documented in centimeters using an anthropometric rod. The dorsal length of the flaccid penis, extending from the pubo-penile junction to the glans' meatus, was measured in millimeters using disposable, malleable, Blayco® (TELIC SAU, Spain) rulers (Fig. 5). The same ruler model was subsequently utilized in multiple steps of the dissection protocol.

### Dissection Protocol

**Fig. 6:**
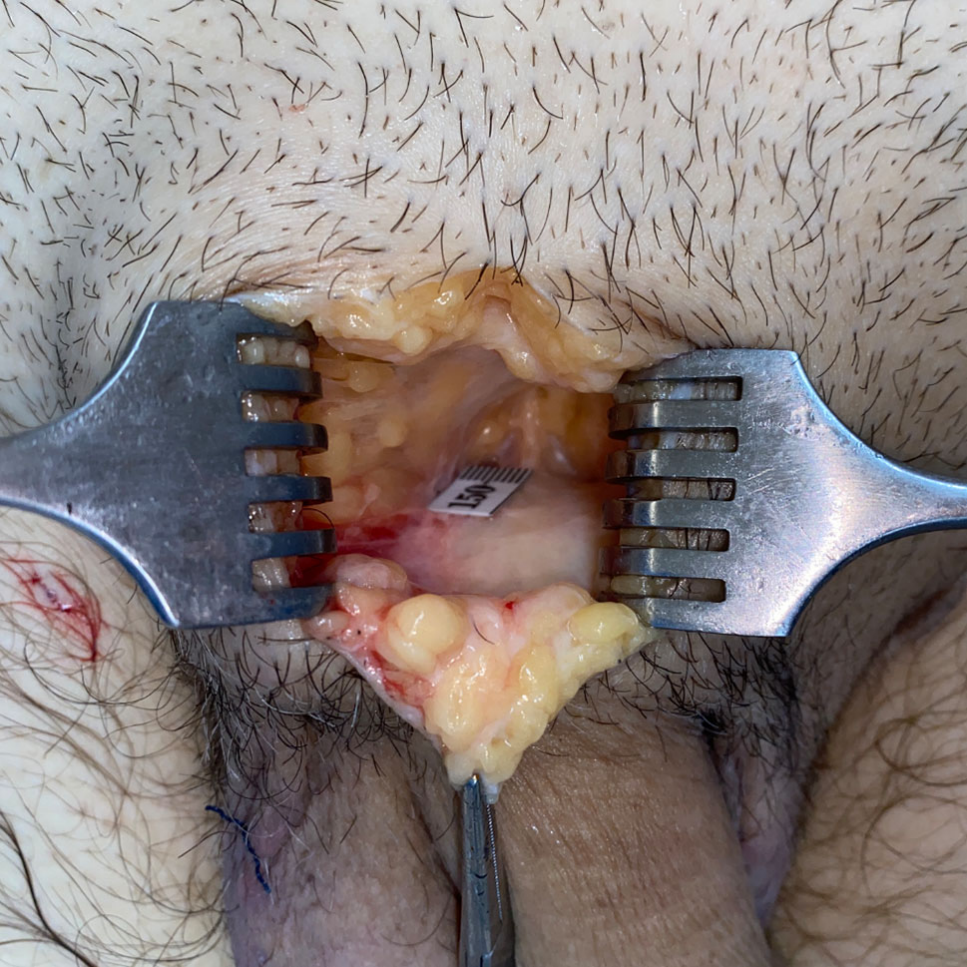
The suspensory ligament of penis is exposed, following careful dissection of the subcutaneous tissue of the abdomen and the membranous layer of abdominal subcutaneous tissue. A piece of disposable measuring tape was placed next to the anterior border of the ligament

**Fig. 7:**
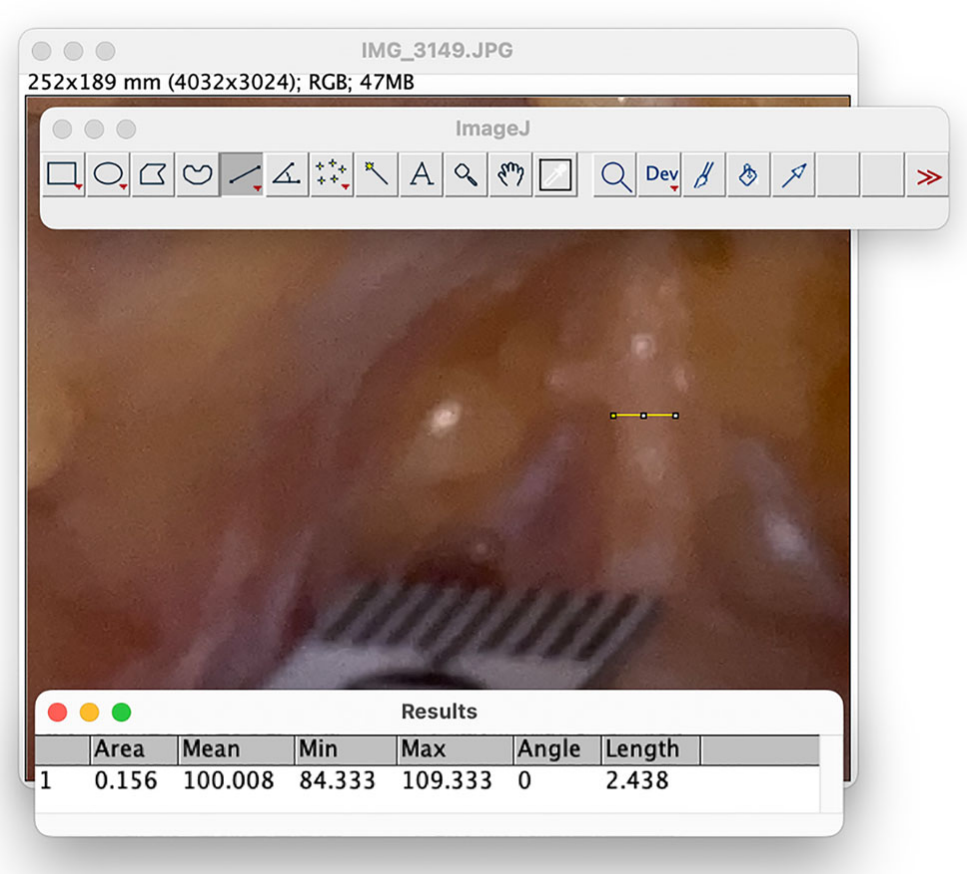
Using the ImageJ program to measure the width of the suspensory ligament of penis

**Fig. 8:**
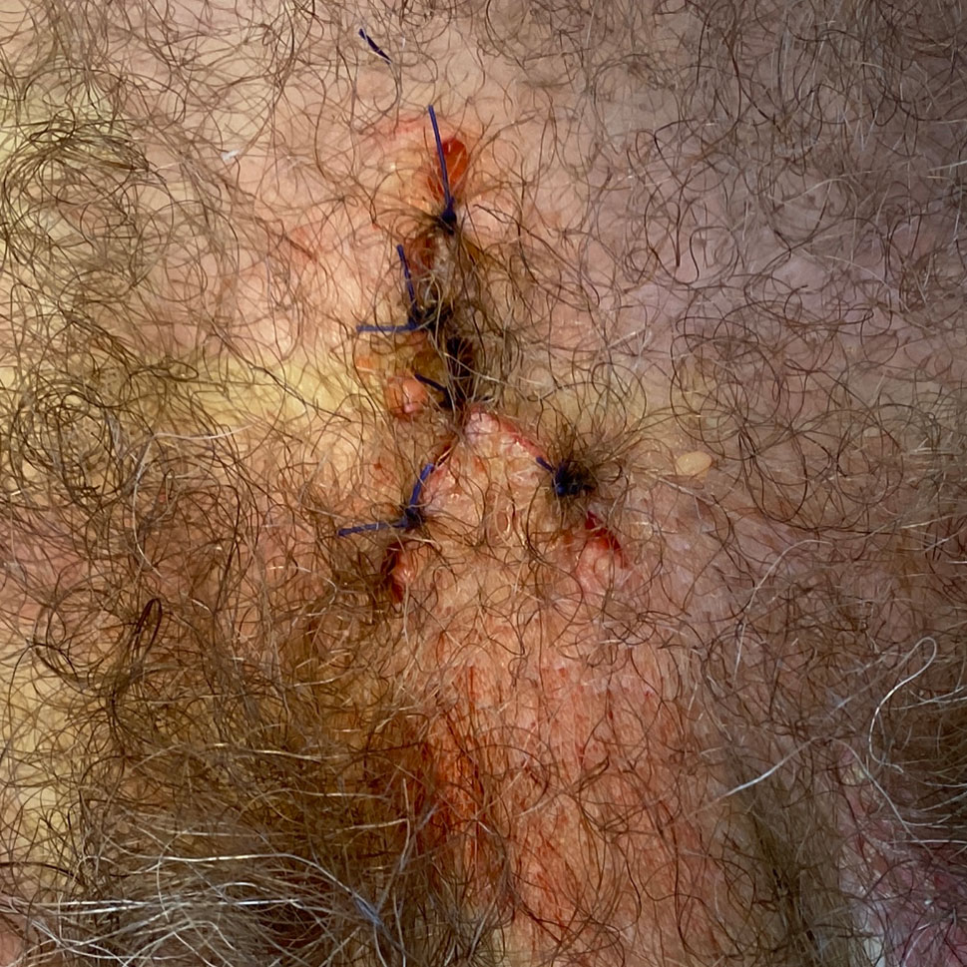
The closed incisions, following the V-Y advancement skin advancement flap

A strategically placed inverted V incision was made at the pubo-penile junction, with the apex directed superiorly. The subcutaneous tissue of the abdomen and the membranous layer of abdominal subcutaneous tissue were meticulously dissected, followed by division of the fundiform ligament. The suspensory ligament of the penis was then exposed. A photograph of the ligament was captured with an Apple® iPhone® 11 (Apple Inc., United States of America) along with a segment of the ruler placed parallel to the ligament to establish a scale. The width of the ligament was measured in millimeters employing the ImageJ (version 1.4.3.67) program developed by the National Institutes of Health (Figs. 6 and 7). The suspensory ligament of the penis was totally dissected up to the inferior border of the pubic symphysis, and the depth of the pubic symphysis was measured in millimeters using a probe and the ruler. The superficial layers of the dissected ligaments were then inverted and sutured to the deepest portion of the pubic periosteum with 3-0 polyglycolic acid suture (VICRYL® 3-0 suture, violet, braided, 45 cm, cutting), in order to fill the dead space created by the division of the suspensory ligament—in vivo, this step would prevent paradoxical penile shortening by reattachment of the penile corpora in a more posterior position of the pubic symphysis. A V-Y advancement flap was utilized to suture the pubic incisions without tension, preventing penile advancement from being hindered by inadequate skin coverage at the pubo-penile junction (Fig. 8). The surgical wound was meticulously closed in two layers—skin and subcutaneous planes—with 4-0 polyglycolic acid suture (VICRYL RAPID™ 4-0 suture, undyed, braided, 45 cm, cutting).

**Fig. 9:**
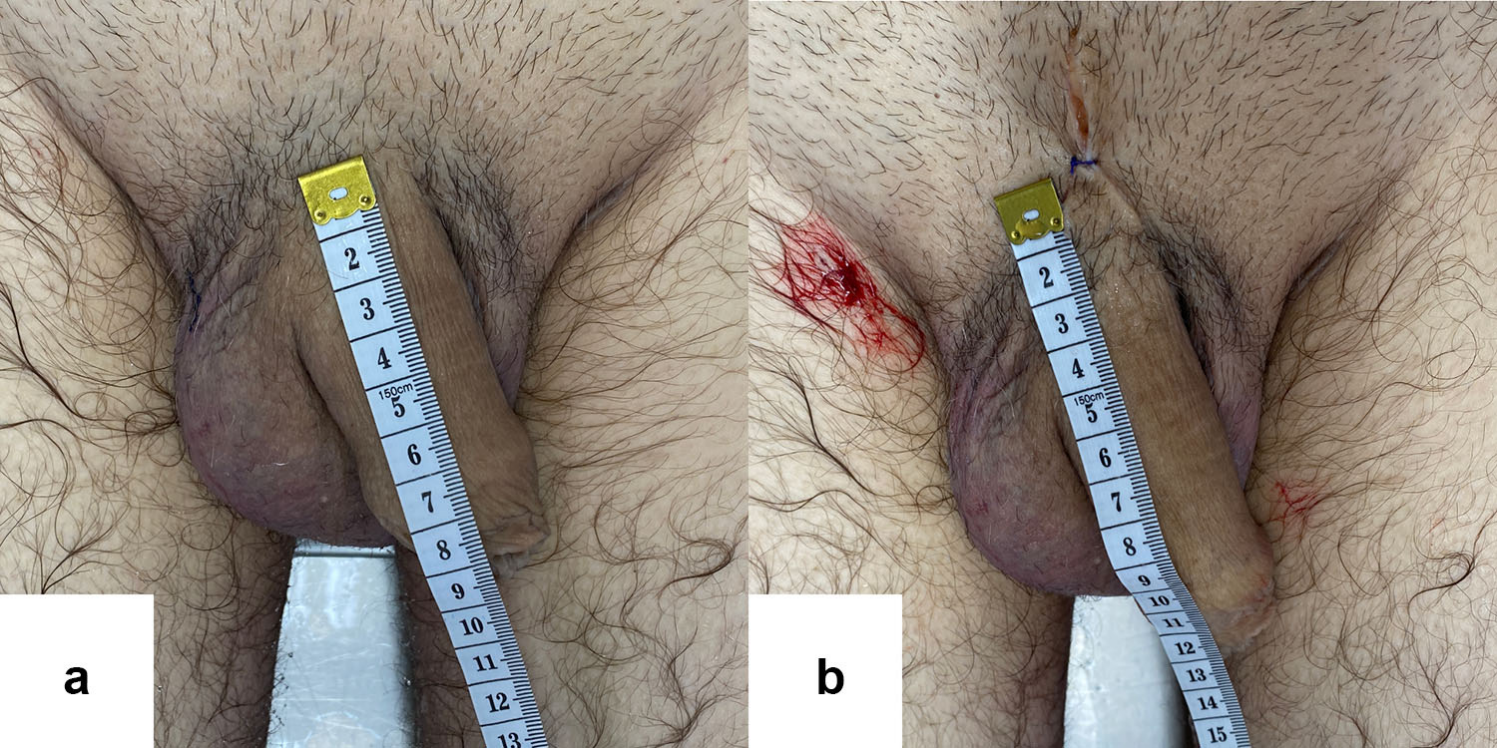
Pre (**a**) and postoperative (**b**) photographs of one of the subjects. Notice the V-Y skin plasty incision located just above the penis in picture **b**. For this subject: pre-ligamentolysis penile length = 90 mm, post-ligamentolysis penile length = 120 mm, absolute penile length increase = 30 mm and relative penile length increase = 33%

**Fig. 10:**
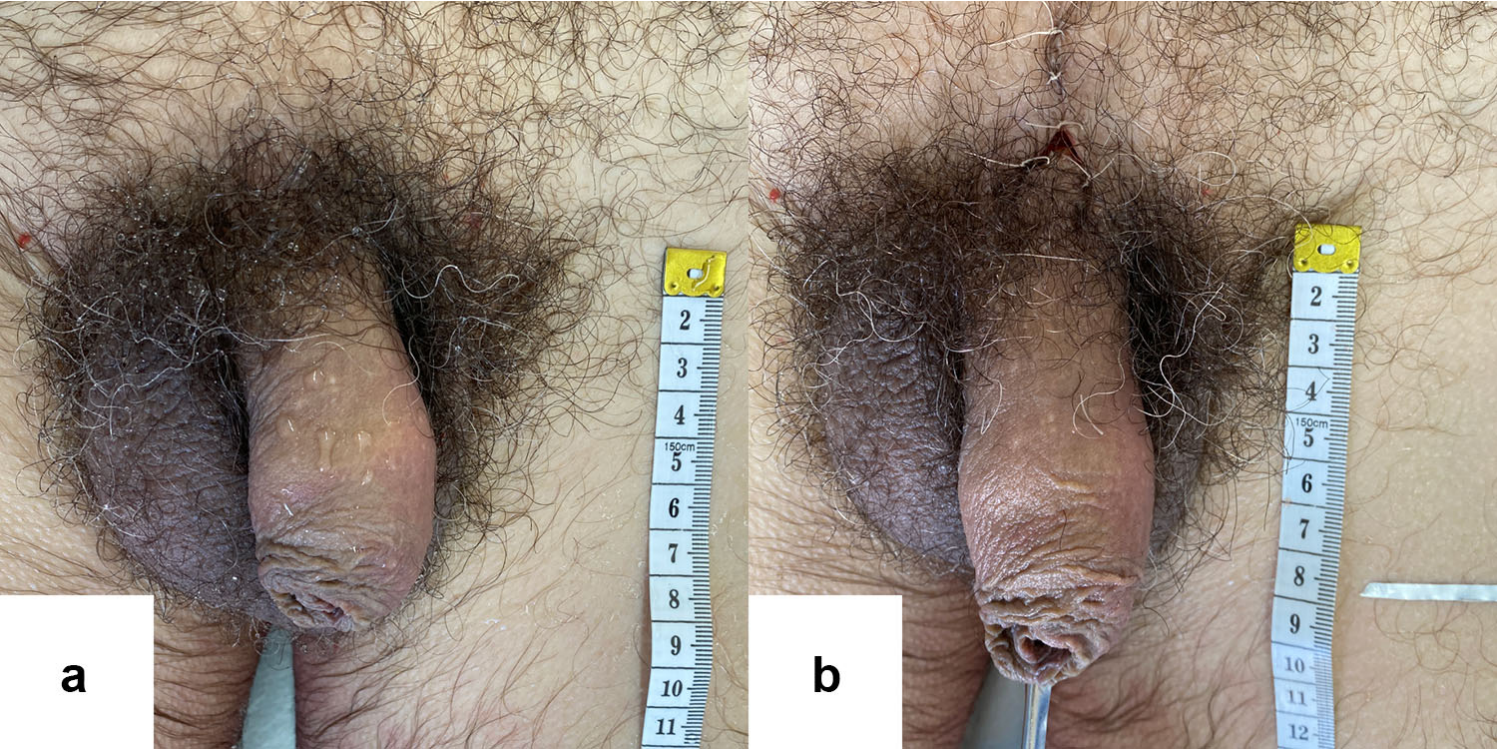
Pre (**a**) and postoperative (**b**) photographs of one of the subjects. For this subject, pre-ligamentolysis penile length = 80 mm, post-ligamentolysis penile length = 100 mm, absolute penile length increase = 20 mm and relative penile length increase = 25%.

**Fig. 11:**
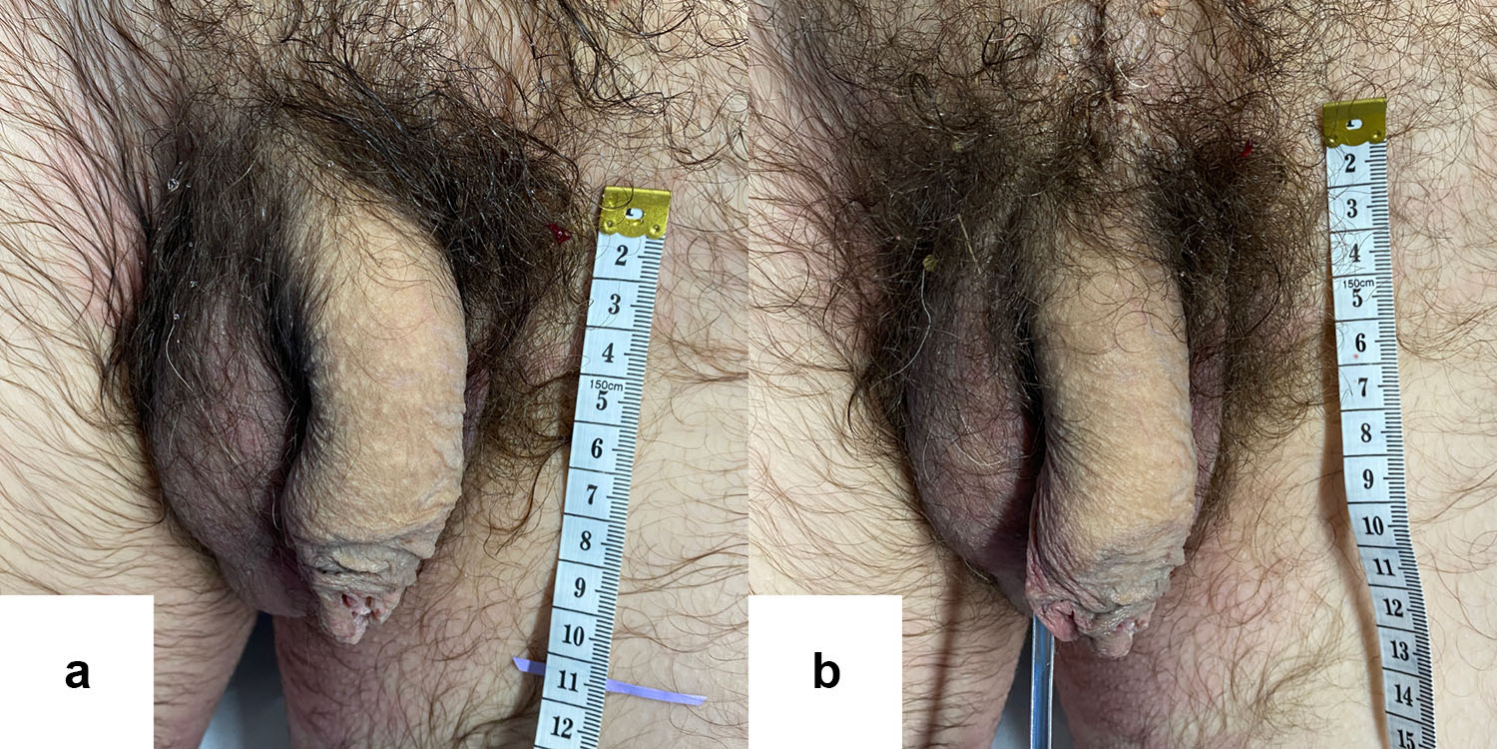
Pre (**a**) and postoperative (**b**) photographs of one of the subjects. For this subject, pre-ligamentolysis penile length = 120 mm, post-ligamentolysis penile length = 145 mm, absolute penile length increase = 25 mm and relative penile length increase = 21%.

Figures 9, 10 and 11 portray images of three subjects before and after the dissection protocol.

### Post-dissection Measurements

The dorsal length of the flaccid penis, extending from the pubo-penile junction to the glans' meatus, was remeasured in millimeters using the ruler. The absolute and relative differences between the flaccid penis length before and after ligamentolysis were calculated and documented in millimeters and percentage, respectively.

### Statistical Analysis

Statistical analyses were conducted using IBM SPSS® Statistics software, version 27. A significance level of *p* ≤ 0.05 was adopted for all statistical inferences. Continuous variables were presented as mean ± standard deviation (SD), and categorical variables were presented as absolute and relative frequencies. To ensure the suitability of parametric tests and correlation coefficients, the normal distribution of continuous variables was evaluated using the Shapiro-Wilk test. All continuous variables exhibited a normal or near-normal distribution. Correlations between the absolute difference in penile length post-ligamentolysis and relevant variables were established using the Pearson correlation coefficient (*r*). The independent-samples *t* test was employed to examine the distribution of the absolute difference in penile length post-ligamentolysis across ethnicities.

## Results

The study sample comprised 16 participants with a mean age at death of 64.38 years (SD = 13.11) (Table 3). Most participants were White (87.50%), with the remaining identifying as Black (12.50%). Anthropometric measurements revealed an average height of 167.19 cm (SD = 5.53), suspensory ligament width of 1.78 mm (SD = 0.73), pubic arch depth of 60.20 mm (SD = 14.04), pre-ligamentolysis penile length of 103.63 mm (SD = 26.03), and post-ligamentolysis length of 130.00 mm (SD = 20.82). The mean absolute size increases after ligamentolysis was 26.38 mm (SD = 14.83; range 4–60).

**Table 3:** Descriptive analysis. SD, standard deviation

Variables	(*n* = 16)
Age at death, mean (SD)	64.38 (13.11)
Ethnicity, *n* (%)	
Black	2 (12.50)
White	14 (87.50)
Height (cm)	167.19 (5.53)
Width of the suspensory ligament (mm), mean (SD)	1.78 (0.73)
Depth of the pubic arch (mm), mean (SD)	60.20 (14.04)
Penile length before ligamentolysis (mm), mean (SD)	103.63 (26.03)
Penile length after ligamentolysis (mm), mean (SD)	130.00 (20.82)
Absolute size increase (mm), mean (SD)	26.38 (14.83)

Pearson correlation analysis revealed a significant negative correlation between pre-ligamentolysis penile length and absolute penile length increase after ligamentolysis (*r* = − 0.601; *p* = 0.014) (Table 4). This indicates that individuals with shorter pre-ligamentolysis penile lengths experienced greater gains in penile length following ligamentolysis. Age, suspensory ligament width, and pubic arch depth did not show significant correlations with absolute length increase. Additionally, the distribution of penile length increase after ligamentolysis did not differ significantly by ethnicity (*t* = − 0.135; *p* = 0.894).

**Table 4:** Pearson correlation coefficient (*r*) between absolute size increase in penile length after ligamentolysis and demographic and anatomical variables

Variables	Correlation coefficient	*P* value
Age at death	−0.173	0.523
Height	0.142	0.599
Penile length before ligamentolysis	−0.601	0.014
Width of the suspensory ligament	−0.420	0.119
Depth of the pubic arch	0.241	0.388

## Discussion

This article delves into a procedure aimed at relocating a section of the concealed posterior (root) of the penis to its visible and movable anterior part (body). This adjustment enables the penis to achieve greater proximity to its erect length even when flaccid, thereby creating the illusion of increased length. Although the absolute size of the penis and its three components (root, body, and glans) remain unchanged, the perceived increase in penile length is more pronounced in its flaccid state [12].

Comprehending the complex anatomy of the penile suspensory system is crucial within the context of this surgical procedure. Due to heterogeneous anatomical descriptions in the literature, a complete yet concise review is necessary. The suspensory apparatus of the penis comprises three main components, which are, from anterior to posterior: the fundiform ligament of the penis, the suspensory ligament of the penis and the inferior pubic ligament [24–26]. They are represented in Figs. 1, 2 and 3.

The fundiform ligament adheres to the posterior surface of the subcutaneous tissue of the anterior abdominal wall. It separates into two paramedian bundles that approach and encircle the perimeter of the corpora cavernosa, without direct connection to the tunica albuginea or the pubic symphysis. At the ventral surface of the corpus spongiosum, the two bundles come together and form the superior portion of the septum of the scrotum. It is independent from the other two penile ligaments, and its role is to support the pendulous part of the penis in front of the pubis. It plays a limited role in erection and is often partially dissected in penile elongation surgery to access the suspensory ligament [8, 20, 26].

The suspensory ligament of penis is located between the pubic symphysis and the corpora cavernosa. Posteriorly, it adheres to the inferior pubic ligament. Its superior margin adheres to the pubic symphysis, and due to this attachment, one can infer that the length of the suspensory ligament proper corresponds to the anteroposterior dimension of the pubic symphysis. Inferiorly, it attaches to the tunica albuginea of the corpora cavernosa. It keeps the root of the penis attached to the pubic symphysis, concealing it and being the main target for penile lengthening surgery.

The inferior pubic ligament is situated between the inferior pubic ramus and the tunica albuginea of the corpora cavernosa. It attaches to the posteroinferior margin of the pubic symphysis superiorly and to the posterior margin of the suspensory ligament of penis anteriorly. Inferiorly it attaches to the tunica albuginea of the corpora cavernosa. The inferior pubic ligament serves as the most robust point of attachment between the penis and the pubic symphysis, playing a crucial role in maintaining penile stability. It is rarely approached in penile lengthening surgery [26, 27].

The dissection of the penile suspensory ligament followed by V-Y skin advancement flap is not a procedure without risks, as summarized in Table 2. It necessitates thorough preoperative evaluation, preparation, and postoperative care to ensure optimal results. Generally, 2 days before surgery, the patient undergoes pubic trichotomy. On the eve and day of the surgery, they should shower and scrub the genital region with chlorhexidine. Post-procedure, patients are advised, in addition to standard surgical wound care and antibiotic prophylaxis, to refrain from sexual activity for approximately 6 weeks, avoid strenuous physical activity for around 1 month, and utilize a penile vacuum/extensor from days 60 to 180 post-op.

Yet, as previously mentioned, this surgery yields highly variable results. Prior knowledge of these outcomes could be invaluable for patients considering surgery, aiding their decision-making process. Surgeons, armed with this information, can offer more informed counseling to patients seeking this procedure.

The results of this study indicate that it was effective in achieving penile lengthening in all individuals, with an average length gain of 26.38 ± 14.83 mm and a range of 4–60 mm. This is in line with the results obtained in previous studies [19, 20, 25, 28, 29].

In this study, it was also observed that subjects with shorter pre-ligamentolysis penile length experienced greater penile length gains following the procedure, which was never described before in the literature.

Although this study identified anatomical factors influencing surgical outcomes, even individuals with less favorable conditions showed some improvement. However, limitations exist due to a small and homogenous sample size, potentially exaggerating age and ethnicity-related effects. Additionally, this study was conducted on cadavers, and might not wholly reflect results in living subjects.

Further comprehensive studies examining the complete length of penile components and their relations with adjacent anatomical structures, potentially utilizing imaging techniques such as ultrasound and conventional X-ray, are imperative. A broader clinical follow-up study is advised, potentially integrating new variables like body fat percentage, to substantiate these discoveries.

## Conclusion

This study demonstrates that the penile lengthening technique of suspensory ligamentolysis, combined with a V-Y advancement flap, consistently produces some degree of penile lengthening, regardless of the individual's intrinsic characteristics. The strong negative correlation between pre-ligamentolysis penile length and absolute penile length increase after ligamentolysis indicates a potential predictability for this procedure. However, further research involving larger and more diverse clinical trials with live patients is necessary to establish more robust and statistically significant correlations and formulas that can be confidently applied in clinical practice.
